# Analysis of risk factors for latent tuberculosis infection among type 2 diabetics: a hospital-based multicenter cross-sectional study

**DOI:** 10.3389/fcimb.2026.1692527

**Published:** 2026-03-24

**Authors:** Zhenyang Liu, Ao Huang, Zhikai Ding, Jiarong Xu, Yawei Cui, Yunxia Wang, Wenzhi Cai

**Affiliations:** 1 Department of Tuberculosis Prevention and Control, Shenzhen Bao’an Center for Chronic Disease Control, Shenzhen, China; 2 Department of Nursing, Shenzhen Hospital, Southern Medical University, Shenzhen, China; 3 School of Nursing, Southern Medical University, Guangzhou, China; 4 Department of Lung Disease, Shenzhen Baoan District People’s Hospital, Shenzhen, China

**Keywords:** infectious disease, interferon-gamma release assay, latent tuberculosis, risk factors, type 2 diabetes mellitus

## Abstract

**Introduction:**

Type 2 diabetes mellitus (T2DM) increases the incidence of active tuberculosis (TB). This study aimed to assess the prevalence and risk factors of latent tuberculosis infection (LTBI) among T2DM in Shenzhen, China.

**Methods:**

A total of 1,363 patients with T2DM and no prior history of TB were enrolled from three tertiary general hospitals. The interferon-gamma release assay (IGRA) was conducted to assess LTBI. Simple and multiple logistic regression analyses were conducted to identify the risk factors related with LTBI among type 2 diabetics. Adjusted odds ratios (aOR) and 95% confidence intervals (CIs) between T2DM and LTBI were estimated using multiple logistic regression, which also corrected for possible covariates.

**Results:**

The prevalence of LTBI among type 2 diabetics was 63.7%. No notable differences were observed in socio-demographic characteristics between LTBI and non-LTBI subjects. Identified risk factors for LTBI included higher education levels, living with a relative with TB, an HbA1c level above 7%, and a hemoglobin level exceeding 14 g/dL.

**Discussion:**

More than half of patients with T2DM are affected by LTBI. Specific environmental exposures and poor glycemic control—evidenced by glycosylated hemoglobin (HbA1c) levels exceeding 7% and hemoglobin levels above 14 g/dL—have been recognized as significant risk factors for LTBI in type 2 diabetics.

## Introduction

The association between diabetes mellitus (DM) and latent tuberculosis infection (LTBI) has garnered significant attention in recent years (Ssekamatte et al., 2024). DM is a complex, multifactorial disease with type 2 diabetes mellitus (T2DM) being the most prevalent form (Shapiro et al., 2025). Individuals with DM have a threefold increased risk of developing tuberculosis (TB), suggesting a shared high inflammatory immune response between those with TB and those with DM (Zhao et al., 2013). DM significantly influences TB pathogenesis and immune responses, and elevated blood glucose levels in TB patients are associated with reduced innate immune cell counts, impaired phagocytic function, and delayed antigen presentation (Goletti et al., 2018; Christian Ngama et al., 2025). According to the World Health Organization (WHO), approximately one-quarter of the global population is asymptomatically infected with mycobacterium tuberculosis (MTB), a condition known as LTBI (Earp et al., 2025; Singh et al., 2025). Individuals with LTBI are at a significantly higher risk of developing active TB (Römpp et al., 2025).

It is widely acknowledged that cellular immunity plays a central role in preventing MTB infection. This process primarily involves the production of interferon-gamma (IFN-γ) and the activation of type I immune responses, which are considered key biomarkers for TB prevention (Felixberger et al., 2025; Van Roy et al., 2025). The interferon-gamma release assay (IGRA) is a modern immunological tool used to diagnose TB infection (Fragoulis et al., 2022). Compared to conventional diagnostic methods, IGRA demonstrates significantly higher sensitivity and specificity (Gupta et al., 2019). A study was conducted among type 2 diabetics to assess the prevalence and predictors of LTBI and to evaluate the diagnostic consistency between the tuberculin skin test (TST) and the IGRA (Abdul-Ghani et al., 2024). In recent years, the prevalence of tuberculosis-diabetes mellitus (TB-DM) comorbidity has increased significantly (Peize et al., 2024), However, systematic studies examining treatment outcomes and influencing factors in TB-DM patients remain limited (Jiang et al., 2022). A deeper understanding of the risk factors associated with LTBI in diabetic patients is essential for developing effective prevention and management strategies.

To investigate the association between LTBI and T2DM, we conducted a hospital-based cross-sectional study to identify risk factors associated with LTBI among adult T2DM patients in China. The study was conducted at the Baoan Center for Chronic Disease Control, Shenzhen Hospital, Southern Medical University, and the Baoan District People’s Hospital in Shenzhen, China.

## Methods

### Study design and participants

This study was conducted from January 2017 to January 2018 at three tertiary general hospitals in Baoan District, Shenzhen, China. These hospitals are recognized as key centers for healthcare, education, and research, each equipped with a capacity of at least 500 beds. This cross-sectional study utilized a convenience sampling method and enrolling consecutive patients diagnosed with T2DM who were receiving healthcare services at the three participating hospitals. The diagnosis of T2DM was established in accordance with the criteria set forth by the American Diabetes Association (ADA). Patients with T2DM received standard antidiabetic medication given by their primary care physician, with metformin being the predominant supplemental oral hypoglycemic drug in our cohort study. The utilization of metformin was not a selection nor an inclusion criterion, and individuals using alternative antidiabetic therapies were not excluded from the analysis.

We compiled a list of eligible subjects based on diagnosis and randomly selected T2DM patients using a computer-generated list of random numbers. Clinical and laboratory tests were conducted to exclude patients with active TB. Patients who declined participation were replaced using the same list and randomization procedure. Health workers visited the homes of the selected participants and adult males and non-pregnant women were invited to participate in the study. As a control group, healthy volunteers including medical staff (administrators, nurses, and physicians) from the three hospitals involved in the study were included. This group, referred to as the non-diabetic group, underwent standardized interviews (Supplementary file 1) and physical examinations to gather clinical, demographic, and LTBI risk factor data.

All subjects underwent laboratory testing to assess the presence of exclusion criteria. The use of immunosuppressive agents or prior treatment for LTBI were considered grounds for exclusion. Additionally, sputum samples and chest radiographs were microscopically examined for potential active TB infection in subjects showing signs or symptoms of active TB. Chest radiographs and sputum samples were reviewed by two experts in the field. Any subjects with findings indicative of active TB or malignancy were excluded from the study.

### Interferon-gamma release assay application and interpretation

The collected blood samples were incubated with MTB-specific antigens, positive control (non-specific stimulant such as phytohemagglutinin) and negative control (culture medium alone). The IGRA utilized MTB-specific peptide antigens from early secretory antigenic target-6 (ESAT-6) and culture filtrate protein-10 (CFP-10) in region of difference 1 (RD1) area, which are not present in BCG strains and most non-tuberculous mycobacteria, hence guaranteeing great specificity for LTBI. IGRA was conducted utilizing fresh whole blood without preceding cell purification. Blood samples were incubated with MTB-specific antigens (ESAT-6 and CFP-10), a nil control, and a mitogen positive control in accordance with the manufacturer’s instructions. Following incubation, the concentration of IFN-γ in the supernatant was quantified by using methods such as enzyme-linked immunosorbent assay (ELISA). A sample was considered positive when the IFN-γ level in the sample after stimulation with MTB-specific antigen is exceeded that of the negative control by a predetermined factor (generally 2-fold or higher) and surpassed a defined threshold. A positive result indicates that the subject may be infected with MTB. Samples stimulated with MTB-specific antigens are considered negative if the IFN-γ level does not significantly increase compared to the negative control, or falls below a defined threshold. However, negative results do not entirely rule out tuberculosis infection as false-negative outcomes may occur due to factors such as early-stage infection, immunocompromised status, or other limitations. In some cases, the result may fall into an indeterminate range between positive and negative, or the interpretation may be challenging due to factors such as sample quality, operational errors, or other technical issues. In such instances, repeat testing or a combination of additional diagnostic methods may be recommended to achieve a comprehensive and accurate assessment.

### Definitions and risk factor assessment

Individuals were categorized into two groups based on their IGRA results: IGRA-positive or IGRA-negative. Venous whole blood samples were collected under fasting conditions on the day of enrollment, prior to IGRA application. Risk factors analyzed via questionnaire and physical examinations included: age, gender, duration of T2DM, glycosylated hemoglobin (HbA1c) levels (categorized as <6.5%, 6.5–7.0%, and >7.0%), metformin use, lymphocyte counts, and peripheral blood neutrophil-to-lymphocyte ratio (NLR) levels. The length of diabetes therapy is indicative of standard clinical practice and differs among individuals. Consequently, treatment duration was not employed as a criterion for stratification or qualifying. Patients diagnosed with LTBI were scheduled for an appointment with a TB specialist, while those without LTBI were scheduled for annual chest X-rays. Anthropometric measurements, including height (in meters), weight (in kilograms), and waist circumference (in centimeters) were taken, while subjects wore light clothing and no shoes. Waist circumference was measured as the maximum circumference at the level of the umbilicus. Body mass index (BMI) was calculated by dividing weight by the square of height. All measurements were performed by trained personnel.

### Sample size calculation and statistical analysis

The sample size was calculated to achieve 80% statistical power at a 5% significance level. As the prevalence of LTBI in the T2DM population is unknown, a prevalence of 50% was assumed for this limited population. This method was selected to optimize variance and guarantee a sufficiently powered sample for the research. Measurement data were expressed as mean ± SD (standard deviation) when normally distributed, and differences between groups were compared using the independent samples t-test or analysis of variance (ANOVA). For non-normally distributed data, results were presented as median (interquartile range), and differences between groups were assessed using the rank sum test. Categorical data were described as frequencies and percentages (%) and group differences were evaluated using the chi-square (*χ²*) test. Variables with a *p*-value of less than 0.25 and those deemed clinically significant were incorporated into the multiple logistic regression analysis. A *p*-value of less than 0.05 and a 95% confidence interval (95% CI) were deemed statistically significant. Data were analyzed using the SPSS version 20.0 statistical software package (SPSS Inc., Chicago, IL, USA).

## Results

### Overview of participant socio-demographic and laboratory characteristics

A total of 1,379 type 2 diabetics were included in this study, of whom 463 (33.6%) were female and 916 (66.4%) were male. Active TB was detected in 16 participants (1.16%), leaving 1,363 subjects for analysis, comprising 455 (33.4%) females and 908 (66.6%) males. The socio-demographic and laboratory characteristics of the study population are delineated in **Table 1**.

**Table 1:** The socio-demographic and laboratory characteristics of type 2 diabetics.

Demographic characteristics	
Age (years) 35.7 ± 14.3	
Gender, n (%)	
Male	908 (66.6)
Female	455 (33.4)
Socioeconomic characteristics	
Educational level, n (%)	
None	439 (32.2)
High school	199 (14.6)
Degree/Master	725 (53.2)
Occupation, n (%)	
Unemployed	595 (43.7)
Employed	706 (51.8)
Student	52 (3.8)
Retired	10 (0.7)
Marital status, n (%)	
Married	1254 (92.4)
Single	95 (6.7)
Divorced	14 (0.9)
Clinical characteristics	
Duration of diabetes (months)	102.4 ± 83.5
Duration of smoking (years)	20.7 ± 1.5
Body mass index (kg/m^2^) Waist circumference (cm)	28.2 ± 6.4 94.8 ± 12.5
Systolic blood pressure (mmHg)	142.7 ± 24.8
Diastolic blood pressure (mmHg)	85.2 ± 12.6
Current smoker, yes, n (%)	368 (26.7)
Alcohol use, yes, n (%)	313 (22.9)
HIV/AIDS, yes, n (%)	3 (0.2)
Laboratory characteristics	
Hemoglobin (g/dL)	14.9 ± 2.7
Leukocytes (per mm^3^)	7156.9 ± 1846.3
Fasting glucose (mg/dL)^a^	164.7 (range 127.4-234.6)
HbA1c (%)	7.8 ± 1.9
Total cholesterol (mg/dL)	197.4 ± 51.8
HDL-C (mg/dL)	46.4 ± 17.0
Triglycerides (mg/dL)^a^	212.0 (range 148.0-304.0)
Serum creatinine (mg/dL)	0.9 ± 0.6

Data are presented as number (percentage), mean ± SD (standard deviation) unless otherwise stated.

HIV, human immunodeficiency virus; AIDS, acquired immune deficiency syndrome; HbA1c, glycosylated hemoglobin; HDL-C, high density lipoprotein cholesterol; n, number.

a Median (25^th^ - 75^th^ percentile).

### Variations in IGRA positivity risk factors and clinical features

A total of 868 (63.7%) type 2 diabetics were tested positive for the IGRA, with a higher prevalence among males (65.7%). **Table 2** summarizes the characteristics of T2DM patients stratified by IGRA reactivity. Systolic (*P* < 0.01) and diastolic (*P* < 0.01) blood pressure were significantly higher in IGRA-negative patients, while hemoglobin levels (*P* < 0.001) were lower in this group. No other significant differences in anthropometric or clinical characteristics were observed between the two groups.

**Table 2:** The clinical and laboratory characteristics of type 2 diabetics according to IGRA reactivity.

Characteristics	IGRA+	IGRA–	*p-*Value
	868	495	
Male, n (%)	570 (65.7)	346 (69.2)	0.26
Age (years)	36.1 ± 14.4	35.1 ± 14.0	0.08
Body mass index (kg/m^2^) Waist circumference (cm) Systolic blood pressure (mmHg)	28.4 ± 6.2 95.6 ± 12.7 140.6 ± 22.6	27.8 ± 6.4 94.6 ± 13.4 144.9 ± 26.7	0.09 0.18 0.01
Diastolic blood pressure (mmHg)	84.3 ± 11.4	85.9 ± 13.2	0.02
Hemoglobin (g/dL)	15.2 ± 1.7	14.2 ± 2.1	<0.001
Leukocytes (per mm^3^) Fasting glucose (mg/dL)^a^	7003.6 ± 1784.7 162 (range127.2-235.8)	7198.2 ± 1896.5 166.8 (range 127.4-233.3)	0.26 0.76^b^
HbA1c (%)	7.6 ± 1.4	7.9 ± 2.3	0.23
Total cholesterol (mg/dL)	198.1 ± 52.3	197.1 ± 51.2	0.65
HDL-C (mg/dL)	46.2 ± 15.9	46.9 ± 16.5	0.50
Triglycerides (mg/dL)^a^	204.2 (range 132.1-274.2)	221.0 (range 152.2-306.9)	0.16^b^
Serum creatinine (mg/dL)	0.8 ± 0.4	0.9 ± 0.7	0.37

Data are presented as number (percentage), mean ± SD (standard deviation) unless otherwise stated.

HbA1c, glycosylated hemoglobin; HDL-C, high density lipoprotein cholesterol; n, number; +, positive;–, negative.

a Median (25^th^ - 75^th^ percentile).

b Mann-Whitney U test.

### IGRA-positive and IGRA-negative type 2 diabetics’ risk factors

**Figure 1:**
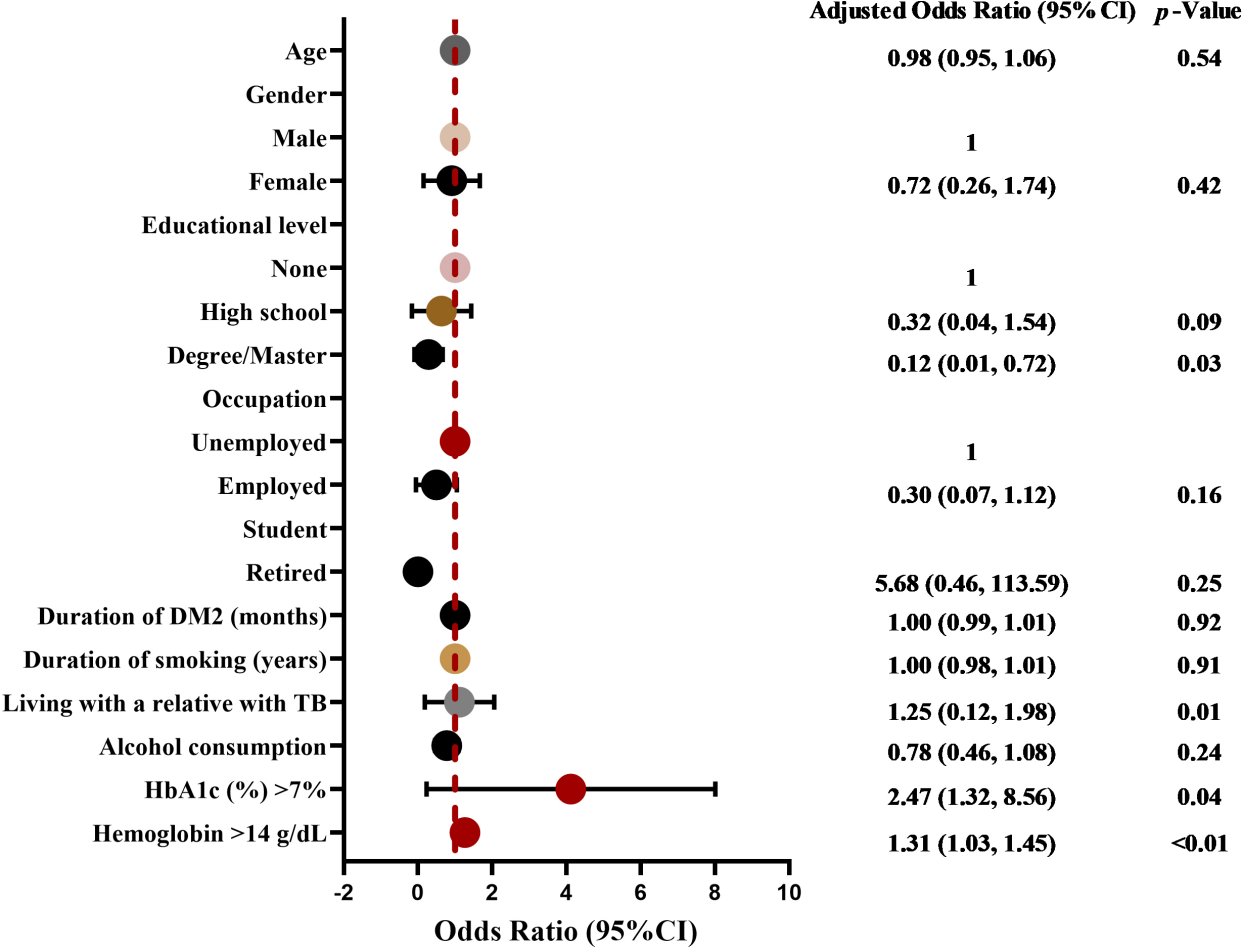
The forest plot of associated factors for LTBI by multiple logistic regression. Variables with a p-value of less than 0.25 and those deemed clinically significant were incorporated into the multiple logistic regression analysis. From the multiple logistic regression analysis, we computed adjusted odds ratios (aORs) and their corresponding 95% confidence intervals (CIs) to evaluate the relationship between type 2 diabetics and LTBI, controlling for potential variables. A *p*-value of <0.05 was considered statistically significant.

**Table 3** illustrates the distribution of risk variables among type 2 diabetics and examines the proportion of individuals in the IGRA-positive and IGRA-negative cohorts. Higher education levels (degree/master/PhD), cohabitation with a relative afflicted by TB, and a Hemoglobin level of 14 g/dL were markedly elevated in individuals with a positive IGRA result. In multivariable logistic regression analysis, elevated HbA1c levels were independently correlated with heightened risks of LTBI following adjustment for possible confounders. Variables exhibiting biological plausibility that demonstrated statistically significant variations among the IGRA groups were employed as independent variables. Consequently, the independent variables include higher education levels (degree/master/PhD), cohabitation with a relative affected by TB, a HbA1c level of 7%, and a hemoglobin level of 14 g/dL, with IGRA serving as the dependent variable. Elevated educational attainment (degree/master/PhD), cohabitation with a relative suffering from TB, a HbA1c level of 7%, and a hemoglobin level of 14 g/dL have been identified as risk factors for LTBI. A notable correlation was identified by multivariate logistic regression between educational attainment and LTBI. Higher education levels (degree/master/PhD) were linked to a higher risk of LTBI (**Figure 1**; **Supplementary Table 1**).

**Table 3:** Risk factors between type 2 diabetics with IGRA-positive and IGRA-negative.

Risk factors	IGRA+		IGRA–		*p-*Value
	n (%)	Mean ± SD	n (%)	Mean ± SD	
Duration of T2DM (months)		103.4 ± 83.5		102.7 ± 83.5	0.75^a^
Duration of smoking (years)		21.3 ± 7.8		20.1 ± 0.4	0.63^a^
Living with a relative with TB	20 (2.3)		7 (1.4)		<0.01
Alcohol consumption		16.3 ± 4.5		14.5 ± 3.8	0.21^a^
Occupation					
Unemployed	404 (46.5)		191 (38.6)		0.46^b^
Employed	424 (48.9)		282 (57.0)		
Student	33 (3.8)		19 (3.8)		
Retired	7 (0.8)		3 (0.6)		
Educational level					
None	253 (29.1)		186 (37.5)		0.19^b^
High school	102 (11.8)		97 (19.6)		
Degree/Master	513 (59.1)		212 (42.8)		
HbA1c (%) >7%	556 (64.2)		257 (52.1)		<0.01
Hemoglobin >14 g/dL	625 (72.4)		317 (63.7)		<0.01

Data are presented as number (percentage), mean ± SD (standard deviation) unless otherwise stated.

T2DM, type 2 diabetes mellitus; TB, tuberculosis;HbA1c, glycosylated hemoglobin; +, positive;–, negative.

a Numerical variable using Independent t-test.

b Categorical variable using Chi-square test.

To further assess the correlation between HbA1c and LTBI risk, HbA1c was stratified into four clinically significant categories (<6.5%, 6.5-6.9%, 7.0-7.9%, and ≥8.0%). Utilizing HbA1c <6.5% as the reference category, patients with HbA1c levels of 7.0-7.9% exhibited elevated chances of LTBI (OR 1.46, 95% CI 1.05-2.02), while those with HbA1c levels of 6.5–6.9% (OR 0.41, 95% CI 0.29-0.57) and ≥8.0% (OR 0.58, 95% CI 0.41-0.82) did not reveal a consistent increase in risk. Trend analysis, using HbA1c categories as an ordinal variable, did not reveal a significant linear correlation (*P* for trend >0.05), indicating a non-linear relationship (**Supplementary Table 2**).

## Discussion

This cross-sectional investigation determined the prevalence of LTBI among type 2 diabetics to be 63.7%. A case-control study conducted in Zhengzhou, China, found a similar prevalence of LTBI among patients with T2DM and healthy controls, as measured by IGRA with a threshold of ≥ 0.35 IU/ml (9.65%) (He et al., 2022). T2DM is a well-established risk factor for developing active TB (Zhou et al., 2023; Guo et al., 2025). Globally, the co-morbidity of T2DM and LTBI is highly prevalent in Africa and Southeast Asia. In Africa, four countries—Uganda, Ethiopia, Nigeria, and Egypt—report a combined T2DM-LTBI co-prevalence of 40% (Kibirige et al., 2023). In Ethiopia, the co-prevalence of T2DM and LTBI is 87% (Smith et al., 2022). China, one of the countries with a high TB burden, has over 200 million individuals infected with LTBI (Ye et al., 2025). In recent years, LTBI screening among diabetic patients has been conducted in various regions of China, with co-infection rates varying significantly due to economic and demographic factors. Generally, LTBI screening in China primarily targets students and healthcare workers, while large-scale studies on diabetic patients remain limited (Zhang et al., 2024).

**Figure 2:**
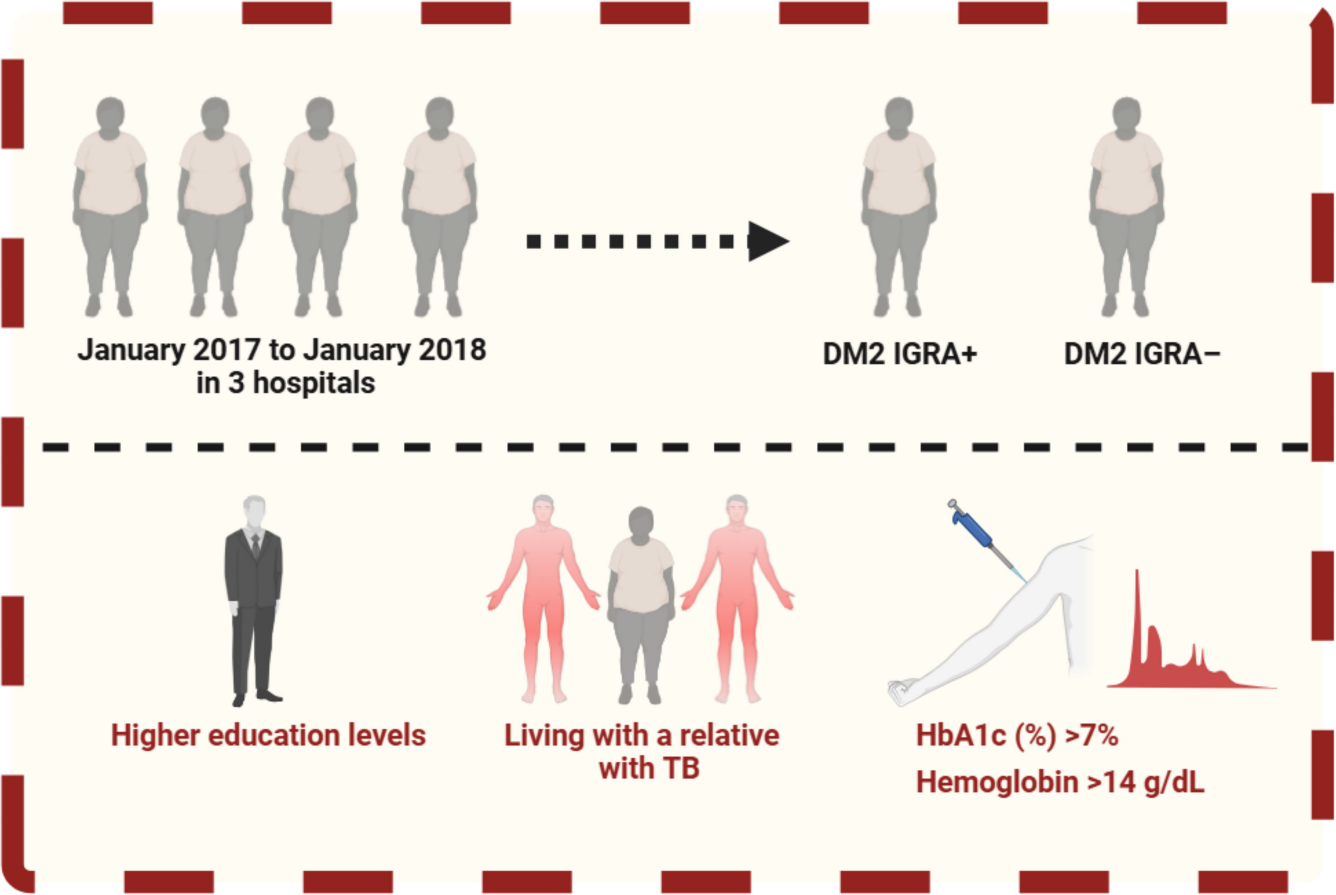
The risk factors for LTBI among type 2 diabetics.

Our investigation revealed that a greater prevalence of LTBI in T2DM correlated with a greater level of education, a finding that contradicts prior research (Ping et al., 2021). The identified correlation between LTBI and T2DM in individuals with elevated educational levels necessitates meticulous analysis. The educational level is improbable to be a direct biological driver of LTBI risk; instead, it may act as a proxy for a range of socioeconomic and behavioral factors. Individuals with higher education may exhibit greater engagement with healthcare systems, experience more regular medical evaluations, or inhabit occupational or urban settings with a larger likelihood of exposure to MTB, thereby elevating the risk of LTBI discovery. The increased health awareness and accessibility to screening may partially account for the elevated prevalence of LTBI found in this cohort. In China, the government and private companies offer a more comprehensive medical insurance system for highly educated individuals, facilitating access to risk screening (Chen et al., 2022). Similarly, among other exposure factors examined in this study, household contacts living with relatives who have TB faced an increased risk of developing active disease, a finding consistent with previous research (Sobota et al., 2016; Ginsburg et al., 2018; Shiferaw et al., 2021). Conversely, no association was found between LTBI and host-dependent risk factors such as age and BMI, aligning with prior studies (Sadirova et al., 2021) (**Figure 2**).

Concerning clinical indicators, two factors correlated with LTBI in individuals with T2DM: HbA1c of 7% and hemoglobin of 14 g/dL (**Figure 2**). A high-glucose environment can impair immune cell function, weaken the host’s ability to combat TB, and increase the risk of MTB infection, ultimately facilitating the progression of LTBI in patients (Kundu et al., 2021). Numerous studies have identified poor glycemic control as a risk factor for LTBI in individuals with DM (Martínez-Aguilar et al., 2015; Huang et al., 2021; Ye et al., 2024). Additionally, impaired MTB-specific T-cell responses have been observed in individuals with diabetes, diagnosed according to the ADA criteria, including HbA1c levels > 7%. A longitudinal study on HbA1c trends during and after TB treatment similarly highlighted the importance of monitoring HbA1c levels in TB patients. In our study, following the adjustment for demographic and clinical variables, inadequate glycemic control was independently linked to the probability of LTBI. Furthermore, poorly controlled T2DM is not only a risk factor for TB reactivation but, as demonstrated in prior studies (Martínez-Aguilar et al., 2015; Bindu et al., 2024; Man-Rong et al., 2024), additionally, it may serve as a risk factor for LTBI and its related consequences. However, elevated hemoglobin is improbable to directly influence susceptibility to LTBI; instead, it may indicate underlying physiological, behavioral, or contextual characteristics that connect with both metabolic condition and infection risk. Higher hemoglobin levels may correlate with male sex, smoking status, occupational exposure, or altitude-related and lifestyle characteristics, all of which have been independently associated with TB exposure or variations in immune response (Ki-Song and Sunmin, 2023). In this case, hemoglobin may serve as a proxy marker rather than a causal agent. Additionally, it may serve as a risk factor for LTBI and its related consequences.

The accuracy of continuous glucose monitoring (CGM) systems in T2DM is closely linked to hemoglobin levels, with variations in accuracy depending on hemoglobin concentration (Rodbard, 2017). A study examining hematological parameters, including hemoglobin levels, in adults with T2DM highlights the importance of monitoring hemoglobin levels in this population (Zhao et al., 2018). Insulin release and activity are essential for glucose homeostasis, and their dysregulation leads to T2DM (Li et al., 2018). Sodium-glucose cotransporter 2 (SGLT2) inhibitors have emerged as a promising therapeutic option for patients with T2DM and coronary artery disease, with benefits attributed to their molecular mechanisms of glucose regulation (Shapiro et al., 2025; Xie et al., 2025). Inadequate glucose control in patients undergoing consistent medical treatment has been extensively documented, underscoring the difficulties of diabetes management in practical environments. Monitoring hemoglobin levels and maintaining them within the normal range are crucial for optimizing T2DM management, with significant implications for cardiovascular health, medication efficacy, and overall patient prognosis.

In this study, participants were screened using the IGRA, which detects TB infection by measuring the number of T-lymphocytes in peripheral blood that release γ-IFN upon stimulation with MTB-specific antigens (Lu et al., 2019). This test relies on a cellular immune response, where T-lymphocytes recognize MTB antigens and produce γ-IFN when the body is infected (Mothé et al., 2015). IGRA offers higher specificity than the traditional TST. The purified protein derivative (PPD) test is influenced by Bacillus Calmette-Guérin (BCG) vaccination and non-tuberculous mycobacterial (NTM) infections, whereas IGRA more effectively distinguishes MTB infections from false-positive reactions caused by these factors (Ruhwald et al., 2017). However, IGRA has certain limitations. As a screening tool for LTBI, it must be supplemented with clinical evaluation and imaging tests for a comprehensive diagnosis. Additionally, IGRA is more expensive than TST, which restricts its widespread use in some regions (Pelzer et al., 2025).

A limitation of this study is that the presence of comorbidities, such as HIV infection, was not assessed, which constitutes a constraint in the study design. Although we initially collected epidemiological data on HIV/AIDS, this information was incomplete due to the small sample size and inconsistencies during patient interviews. Comparably, given the cross-sectional design of this study, establishing causality with certainty is challenging. This data should not be construed as proof of a causative link between higher education and heightened vulnerability to LTBI. Residual confounding from unmeasured socioeconomic attributes, lifestyle factors, or contextual exposures cannot be ruled out. We thus see this connection as hypothesis-generating rather than definitive. Likewise, there is an absence of direct biological interaction evidence linking hemoglobin concentration to the incidence of LTBI in individuals with T2DM. This study was conducted in only three hospitals in southern China, which limits the generalizability of the findings due to significant geographical and cultural variations between northern and southern China, as well as between Eastern and Western regions. Investigations concerning the interplay between T2DM and LTBI remain scarce. Future longitudinal studies must incorporate more comprehensive indicators, including socioeconomic status, healthcare utilization, and exposure history, to elucidate the intricate relationships among education level, T2DM, and LTBI risk. Extensive multicenter epidemiological investigations and randomized controlled trials are essential for enhancing our comprehension in this domain.

## Conclusion

This study revealed a high prevalence of LTBI among patients T2DM in western Shenzhen, China. The primary risk factors associated with LTBI included higher education levels and prolonged exposure to family members with a history of TB. Additionally, poor glycemic control, indicated by HbA1c levels > 7% and hemoglobin levels > 14 g/dL, was identified as a significant risk factor for LTBI in type 2 diabetics. Prospective studies are essential to further examine the temporal association between T2DM and LTBI, as well as the development of illness.

## Data Availability

The original contributions presented in the study are included in the article/**Supplementary Material**. Further inquiries can be directed to the corresponding authors.

## References

[B1] Abdul-Ghani R. Al-Awadi A. Al-Aghbari N. Al-Mikhlafy A. A. Abdulmoghni S. S. Al-Dobai S. S. . (2024). Latent tuberculosis infection and diagnostic performance of the tuberculin skin test among type 2 diabetics in Sana’a city, Yemen. BMC Infect. Dis. 24 (1), 1005. doi: 10.1186/s12879-024-09931-8, PMID: 39300351 PMC11411833

[B2] Bindu D. Anuradha R. Arul Nancy P. Sivakumar S. Sujatha N. Subash B. (2024). Immunological mechanisms of tuberculosis susceptibility in TB-infected individuals with type 2 diabetes mellitus: insights from mycobacterial growth inhibition assay and cytokine analysis. Microbiol. Spectr. 13 (1), e0144524. doi: 10.1128/spectrum.01445-24, PMID: 39656000 PMC11705871

[B3] Chen X. Giles J. Yao Y. Yip W. Meng Q. Berkman L. . (2022). The path to healthy ageing in China: a Peking University-Lancet Commission. Lancet (London England) 400, 1967–2006. doi: 10.1016/S0140-6736(22)01546-X, PMID: 36423650 PMC9801271

[B4] Christian Ngama K. Gift Cilubula M. Serge Kapend M. Michel Muteya M. Harvey Kabulo K. Michel Nzaji K. . (2025). Service availability and readiness of tuberculosis units’ clinics to manage diabetes mellitus in Lubumbashi, Democratic Republic of the Congo. BMC Health Serv. Res. 25 (1), 233. doi: 10.1186/s12913-025-12368-7, PMID: 39934811 PMC11816995

[B5] Earp J. Garaeva A. Meikle V. Niederweis M. Seeger M. (2025). Structural basis of siderophore export and drug efflux by Mycobacterium tuberculosis. Nat. Commun. 16, 1934. doi: 10.1038/s41467-025-56888-6, PMID: 39994240 PMC11850643

[B6] Felixberger P. T. Andrieux G. Maul-Pavicic A. Goldacker S. Harder I. Gutenberger S. . (2025). CD21(low) B cells reveal a unique glycosylation pattern with hypersialylation and hyperfucosylation. Front. Immunol. 16. doi: 10.3389/fimmu.2025.1512279, PMID: 40013136 PMC11861550

[B7] Fragoulis G. E. Nikiphorou E. Dey M. Zhao S. S. Courvoisier D. S. Arnaud L. . (2022). 2022 EULAR recommendations for screening and prophylaxis of chronic and opportunistic infections in adults with autoimmune inflammatory rheumatic diseases. Ann. Rheum Dis. 82 (6), 742–753. doi: 10.1136/ard-2022-223335, PMID: 36328476

[B8] Ginsburg C. Bocquier P. Béguy D. Afolabi S. Kahn K. Obor D. . (2018). Association between internal migration and epidemic dynamics: an analysis of cause-specific mortality in Kenya and South Africa using health and demographic surveillance data. BMC Public Health 18, 918. doi: 10.1186/s12889-018-5851-5, PMID: 30049267 PMC6062880

[B9] Goletti D. Lindestam Arlehamn C. S. Scriba T. J. Anthony R. Cirillo D. M. Alonzi T. . (2018). Can we predict tuberculosis cure? What tools are available? Eur. Respir. J. 52 (5), 1801089. doi: 10.1183/13993003.01089-2018, PMID: 30361242

[B10] Guo J. Feng X. Pang J. Li W. Cai M. Cao Z. . (2025). Risk factors for rifampicin-susceptible and isoniazid-resistant tuberculosis in adult patients with type 2 diabetes mellitus in Nanjing. BMC Infect. Dis. 25, 335. doi: 10.1186/s12879-025-10709-9, PMID: 40065241 PMC11892190

[B11] Gupta R. K. Lipman M. Jackson C. Sitch A. J. Southern J. Drobniewski F. . (2019). Quantitative IFN-γ Release assay and tuberculin skin test results to predict incident tuberculosis. A prospective cohort study. Am. J. Respir. Crit. Care Med. 201 (8), 984–991. doi: 10.1164/rccm.201905-0969OC, PMID: 31825645 PMC7159430

[B12] He Y. Cao X. Guo T. He Y. Du Y. Zhang H. . (2022). Serial testing of latent tuberculosis infection in patients with diabetes mellitus using interferon-gamma release assay, tuberculin skin test, and creation tuberculin skin test. Front. Public Health 10, 1025550. doi: 10.3389/fpubh.2022.1025550, PMID: 36530654 PMC9754324

[B13] Huang H. Huang W. Lin K. Liu S. Lee M. Cheng M. . (2021). Completion rate and safety of programmatic screening and treatment for latent tuberculosis infection in elderly patients with poorly controlled diabetic mellitus: A prospective multicenter study. Clin. Infect. Dis. 73, e1252–e1260. doi: 10.1093/cid/ciab209, PMID: 33677558 PMC8442788

[B14] Jiang W. Trimawartinah Rahman F. M. Wibowo A. Sanjaya A. Silitonga P. I. I. . (2022). The co-management of tuberculosis-diabetes co-morbidities in Indonesia under the National Tuberculosis Control Program: results from a cross-sectional study from 2017 to 2019. BMC Public Health 22 (1), 689. doi: 10.1186/s12889-022-13017-y, PMID: 35395745 PMC8990273

[B15] Kibirige D. Andia-Biraro I. Kyazze A. Olum R. Bongomin F. Nakavuma R. . (2023). Burden and associated phenotypic characteristics of tuberculosis infection in adult Africans with diabetes: a systematic review. Sci. Rep. 13, 19894. doi: 10.1038/s41598-023-47285-4, PMID: 37963989 PMC10645877

[B16] Ki-Song K. Sunmin P. (2023). Impact of lung-related polygenic risk scores on chronic obstructive pulmonary disease risk and their interaction with w-3 fatty acid intake in middle-aged and elderly individuals. Nutrients 15 (13), 3062. doi: 10.3390/nu15133062, PMID: 37447386 PMC10346150

[B17] Kundu J. Verma A. Verma I. Bhadada S. Sharma S. (2021). Mycobacterium tuberculosis withMolecular mechanism of interaction of host macrophages under high glucose conditions. Biochem. biophysics Rep. 26, 100997. doi: 10.1016/j.bbrep.2021.100997, PMID: 33997314 PMC8091876

[B18] Li X. Meng X. Gao X. Pang X. Wang Y. Wu X. . (2018). Elevated serum xanthine oxidase activity is associated with the development of type 2 diabetes: A prospective cohort study. Diabetes Care 41, 884–890. doi: 10.2337/dc17-1434, PMID: 29437822

[B19] Lu L. Smith M. Yu K. Luedemann C. Suscovich T. Grace P. . (2019). IFN-γ-independent immune markers of Mycobacterium tuberculosis exposure. Nat. Med. 25, 977–987. doi: 10.1038/s41591-019-0441-3, PMID: 31110348 PMC6559862

[B20] Man-Rong X. Jun-Wei W. Yi-Lin M. Yu-Jie W. Meng-Han L. Jun-Xi L. . (2024). High-normal serum bilirubin is a useful indicator to assess the risk of diabetic retinopathy in type 2 diabetes: A real-world study. Heliyon 10 (15), e34946. doi: 10.1016/j.heliyon.2024.e34946, PMID: 39157310 PMC11327566

[B21] Martínez-Aguilar G. Serrano C. Castañeda-Delgado J. Macías-Segura N. Hernández-Delgadillo N. Enciso-Moreno L. . (2015). Associated risk factors for latent tuberculosis infection in subjects with diabetes. Arch. Med. Res. 46, 221–227. doi: 10.1016/j.arcmed.2015.03.009, PMID: 25864989

[B22] Mothé B. Lindestam Arlehamn C. Dow C. Dillon M. Wiseman R. Bohn P. . (2015). The TB-specific CD4(+) T cell immune repertoire in both cynomolgus and rhesus macaques largely overlap with humans. Tuberculosis (Edinburgh Scotland) 95, 722–735. doi: 10.1016/j.tube.2015.07.005, PMID: 26526557 PMC4773292

[B23] Peize Z. Huaifang S. Yongping X. Jiemei L. Qiumeng H. Liang F. . (2024). Optimized anti-tuberculosis duration for drug-susceptible pulmonary tuberculosis-diabetes mellitus comorbidities: study protocol for a multicenter randomized controlled trial. BMC Pulm Med. 24 (1), 469. doi: 10.1186/s12890-024-03271-8, PMID: 39334186 PMC11438111

[B24] Pelzer P. Stuck L. Martinez L. Richards A. Acuña-Villaorduña C. Aronson N. . (2025). Effectiveness of the primary Bacillus Calmette-Guérin vaccine against the risk of Mycobacterium tuberculosis infection and tuberculosis disease: a meta-analysis of individual participant data. Lancet Microbe 6, 100961. doi: 10.1016/j.lanmic.2024.100961, PMID: 39709975 PMC12778190

[B25] Ping P. Zakaria R. Islam M. Yaacob L. Muhamad R. Wan Mohamad W. . (2021). Prevalence and risk factors of latent tuberculosis infection (LTBI) in patients with type 2 diabetes mellitus (T2DM). Int. J. Environ. Res. Public Health 18 (1), 305. doi: 10.3390/ijerph18010305, PMID: 33406582 PMC7794868

[B26] Rodbard D. (2017). Continuous glucose monitoring: A review of recent studies demonstrating improved glycemic outcomes. Diabetes Technol. Ther. 19, S25–S37. doi: 10.1089/dia.2017.0035, PMID: 28585879 PMC5467105

[B27] Römpp A. Treu A. Kokesch-Himmelreich J. Marwitz F. Dreisbach J. Aboutara N. . (2025). The clinical-stage drug BTZ-043 accumulates in murine tuberculosis lesions and efficiently acts against Mycobacterium tuberculosis. Nat. Commun. 16, 826. doi: 10.1038/s41467-025-56146-9, PMID: 39827265 PMC11742723

[B28] Ruhwald M. Aggerbeck H. Gallardo R. Hoff S. Villate J. Borregaard B. . (2017). Safety and efficacy of the C-Tb skin test to diagnose Mycobacterium tuberculosis infection, compared with an interferon γ release assay and the tuberculin skin test: a phase 3, double-blind, randomised, controlled trial. Lancet Respir. Med. 5, 259–268. doi: 10.1016/S2213-2600(16)30436-2, PMID: 28159608

[B29] Sadirova D. Grigoryan R. Parpieva N. Barotova V. Trubnikov A. Kalandarova L. . (2021). Incidence rate and risk factors for tuberculosis among people living with HIV: A 2015–2017 cohort from Tashkent, Uzbekistan. Int. J. Environ. Res. Public Health 18, 5746. doi: 10.3390/ijerph18115746, PMID: 34071899 PMC8199393

[B30] Shapiro S. Yin H. Yu O. Rej S. Suissa S. Azoulay L. (2025). Glucagon-like peptide-1 receptor agonists and risk of suicidality among patients with type 2 diabetes: active comparator, new user cohort study. BMJ (Clinical Res. ed.) 388, e080679. doi: 10.1136/bmj-2024-080679, PMID: 40010803 PMC11863255

[B31] Shiferaw M. Sinishaw M. Amare D. Alem G. Asefa D. Klinkenberg E. (2021). Prevalence of active tuberculosis disease among healthcare workers and support staff in healthcare settings of the Amhara region, Ethiopia. PloS One 16, e0253177. doi: 10.1371/journal.pone.0253177, PMID: 34115821 PMC8195404

[B32] Singh D. Ahmed M. Akter S. Shivanna V. Bucşan A. Mishra A. . (2025). Prevention of tuberculosis in cynomolgus macaques by an attenuated Mycobacterium tuberculosis vaccine candidate. Nat. Commun. 16, 1957. doi: 10.1038/s41467-025-57090-4, PMID: 40000643 PMC11861635

[B33] Smith A. Kempker R. Wassie L. Bobosha K. Nizam A. Gandhi N. . (2022). Mycobacterium tuberculosisThe impact of diabetes and prediabetes on prevalence of infection among household contacts of active tuberculosis cases in Ethiopia. Open Forum Infect. Dis. 9, ofac323. doi: 10.1093/ofid/ofac323, PMID: 36420425 PMC9595051

[B34] Sobota R. Stein C. Kodaman N. Scheinfeldt L. Maro I. Wieland-Alter W. . (2016). A locus at 5q33.3 confers resistance to tuberculosis in highly susceptible individuals. Am. J. Hum. Genet. 98, 514–524. doi: 10.1016/j.ajhg.2016.01.015, PMID: 26942285 PMC4800052

[B35] Ssekamatte P. Nabatanzi R. Sitenda D. Nakibuule M. Bagaya B. S. Kibirige D. Kyazze A. P. . (2024). Impaired Mycobacterium tuberculosis-specific T-cell memory phenotypes and functional profiles among adults with type 2 diabetes mellitus in Uganda. Front. Immunol. 15. doi: 10.3389/fimmu.2024.1480739, PMID: 39430752 PMC11486641

[B36] Van Roy Z. Kak G. Fallet R. W. Kielian T. (2025). Interferon-gamma receptor signaling regulates innate immunity during Staphylococcus aureus craniotomy infection. J. Neuroinflamm. 22 (1), 46. doi: 10.1186/s12974-025-03376-9, PMID: 39987156 PMC11847343

[B37] Xie Y. Choi T. Al-Aly Z. (2025). Mapping the effectiveness and risks of GLP-1 receptor agonists. Nat. Med. 31 (3), 951–962. doi: 10.1038/s41591-024-03412-w, PMID: 39833406

[B38] Ye L. Cao Y. Fu Y. Tian C. Cao Q. (2025). Crohn’s disease with latent tuberculosis infection or intestinal tuberculosis: rapid discrimination by targeted next-generation sequencing. Alimentary Pharmacol. Ther. 61, 1218–1225. doi: 10.1111/apt.18522, PMID: 39905821 PMC11908111

[B39] Ye Z. Li L. Yang L. Zhuang L. Aspatwar A. Wang L. . (2024). Impact of diabetes mellitus on tuberculosis prevention, diagnosis, and treatment from an immunologic perspective. Explor. (Beijing China) 4, 20230138. doi: 10.1002/EXP.20230138, PMID: 39439490 PMC11491313

[B40] Zhang L. Li Y. Zou X. Ma H. Gao M. Ge Q. . (2024). Mycobacterium tuberculosisDiagnostic accuracy of -specific triple-color FluoroSpot assay in differentiating tuberculosis infection status in febrile patients with suspected tuberculosis. Front. Immunol. 15, 1462222. doi: 10.3389/fimmu.2024.1462222, PMID: 39845975 PMC11751065

[B41] Zhao W. Shi L. Fonseca V. He J. Shao D. Zhao J. . (2013). Screening patients with type 2 diabetes for active tuberculosis in communities of China. Diabetes Care 36, e159–e160. doi: 10.2337/dc13-1007, PMID: 23970731 PMC3747870

[B42] Zhao L. Zhang F. Ding X. Wu G. Lam Y. Wang X. . (2018). Gut bacteria selectively promoted by dietary fibers alleviate type 2 diabetes. Sci. (New York N.Y.) 359, 1151–1156. doi: 10.1126/science.aao5774, PMID: 29590046

[B43] Zhou G. Guo X. Cai S. Zhang Y. Zhou Y. Long R. . (2023). Diabetes mellitus and latent tuberculosis infection: an updated meta-analysis and systematic review. BMC Infect. Dis. 23, 770. doi: 10.1186/s12879-023-08775-y, PMID: 37940866 PMC10631079

