# Supplementary material for: Analysis of risk factors for latent tuberculosis infection among type 2 diabetics: a hospital-based multicenter cross-sectional study
Source: Front Cell Infect Microbiol. 2026 Mar 24;16:1692527. doi: 10.3389/fcimb.2026.1692527 (PMC13055539; doi:10.3389/fcimb.2026.1692527)
Supplement: Supplementary file 1 [file Table1.docx]

**Table S1**. Associated factors for LTBI by multiple logistic regression.

| **Factors** | **Regression**  **Coefficient** | **Adjusted** **Odds**  **Ratio (95% CI)** | ***p-*Value** |
| --- | --- | --- | --- |
| Age | −0.140 | 0.98 (0.95, 1.06) | 0.54 |
| **Gender** |  |  |  |
| Male |  | 1.0 |  |
| Female | −0.374 | 0.72 (0.26, 1.74) | 0.42 |
| **Educational level** |  |  |  |
| None |  | 1.0 |  |
| High school | −1.358 | 0.32 (0.04, 1.54) | 0.09 |
| Degree/Master | −2.623 | 0.12 (0.01, 0.72) | 0.03 |
| **Occupation** |  |  |  |
| Unemployed |  | 1.0 |  |
| Employed | −1.172 | 0.30 (0.07, 1.12) | 0.16 |
| Student | 1.842 | 5.68 (0.46, 113.59) | 0.25 |
| Retired | −17.943 | 0.000 | >0.95 |
| Duration of DM2 (months) | 0.000 | 1.00 (0.99, 1.01) | 0.92 |
| Duration of smoking (years) | 0.000 | 1.00 (0.98, 1.01) | 0.91 |
| Living with a relative with TB | −0.167 | 1.25 (0.12, 1.98) | 0.01 |
| Alcohol consumption | −0.562 | 0.78 (0.46, 1.08) | 0.24 |
| HbA1c (%) >7% | −0.026 | 2.47 (1.32, 8.56) | 0.04 |
| Hemoglobin >14 g/dL | −0.054 | 1.31 (1.03, 1.45) | <0.01 |

*Note*: Data are presented as number (percentage), mean ± SD (standard deviation) unless otherwise stated.

Abbreviations: DM2，type 2 diabetes mellitus; TB, tuberculosis；HbA1c, glycosylated hemoglobin.
