# Supplementary material for: Analysis of risk factors for latent tuberculosis infection among type 2 diabetics: a hospital-based multicenter cross-sectional study
Source: Front Cell Infect Microbiol. 2026 Mar 24;16:1692527. doi: 10.3389/fcimb.2026.1692527 (PMC13055539; doi:10.3389/fcimb.2026.1692527)
Supplement: Supplementary file 2 [file Table2.docx]

|  | **IGRA+** | **IGRA–** | **Odds Ratio (95% CI)** | ***P*** |
| --- | --- | --- | --- | --- |
| HbA1c (%) | 868 | 495 |  |  |
| Total |  |  |  |  |
| <6.5% | 179 | 84 |  |  |
| 6.5–6.9% | 133 | 154 | 0.41 (0.29, 0.57) | *P* < 0.001 |
| 7.0–7.9% | 395 | 127 | 1.46 (1.05, 2.02) | 0.02 |
| ≥8.0% | 161 | 130 | 0.58 (0.41, 0.82) | 0.002 |

**Table S2**. Stratified analysis and trend test of HbA1c

*Note*: Using <6.5% as a reference, crude (unadjusted) Logistic regression OR and 95% CI. When performing a linear trend test by assigning values of 1-4 to the four categories of HbA1c, no significant linear trend was observed (*P* > 0.05).

Abbreviations: HbA1c, glycosylated hemoglobin.
